# Arousing emoticons edit stream/bounce perception of objects moving past each other

**DOI:** 10.1038/s41598-018-23973-4

**Published:** 2018-04-10

**Authors:** Akihiko Gobara, Naoto Yoshimura, Yuki Yamada

**Affiliations:** 1 Graduate School of Human-Environment Studies, Kyushu University, Fukuoka, Japan; 2 Japan Society for the Promotion of Science, Tokyo, Japan; 3 Faculty of Arts and Science, Kyushu University, Fukuoka, Japan

## Abstract

When two identical objects move toward each other, overlap completely, and continue toward opposite ends of a space, observers might perceive them as streaming through or bouncing off each other. This phenomenon is known as ‘stream/bounce perception’. In this study, we investigated the effect of the presentation of emoticons on stream/bounce perception in five experiments. In Experiment 1, we used emoticons representing anger (‘(‘∧’)’), a smile (‘(^_^)’), and a sober face (‘(°_°)’, as a control), and observers were asked to judge whether two objects unrelated to the emoticon had streamed through or bounced off each other. The anger emoticon biased perception toward bouncing when compared with the smile or sober face emoticon. In Experiments 2 and 3, we controlled for the valence and arousal of emoticons, and found that arousal influenced stream/bounce perception but valence did not. Experiments 4 and 5 ruled out the possibility of attentional capture and response bias for the emoticon with higher arousal. Taken together, the findings indicate that emoticons with higher arousal evoke a mental image of a ‘collision’ in observers, thereby eliciting the bounce perception.

## Introduction

When two identical objects located at opposite corners of a space move toward each other, overlap completely, and pass toward opposite the sides of a space, observers can perceive the objects as either streaming through or bouncing off each other. This bistable visual phenomenon is known as ‘stream/bounce perception’^1^. Generally, the streaming perception is dominant in stream/bounce perception, although no comprehensive mechanism for this predominance has been proposed yet (for discussions^2–4^).

Several features of moving objects can bias stream/bounce perception toward bouncing. First, an additional auditory cue at or near the coincidence can alter stream/bounce perception. Sekuler, Sekuler, and Lau^5^ found that the proportion of trials in which participants perceived bouncing increased when a brief click was presented around the coincidence of the moving objects. The effect of sound appears to have a temporal window of ±100 ms from the coincidence although this temporal window is asymmetrical^6^. Another study^7^ reported that a decaying sound can induce bouncing, whereas a ramping sound cannot, thus indicating that the effect of sound on stream/bounce perception depends on the characteristics of the sound. Moreover, further investigation^8^ of this issue by manipulating the contextual information of the sounds presented the sound of a drop of water, a collision of billiard balls, or a firework such that the intensity peaked at ~200 ms before the moving objects’ overlap. The results showed that all three sound cues biased perception toward bouncing, with the strongest bias being found for the collision of billiard balls. This finding suggests that bounce-congruent sounds strongly bias perception toward bouncing, meaning that the contextual information of a sound cue plays an important role in the modulation of stream/bounce perception. Relevantly, studies using signal detection theory (SDT) have revealed that other sounds can influence the decision criterion in stream/bounce perception, but not the actual sensory process^9,10^ (but see^11^). A functional magnetic resonance imaging (fMRI) study suggests that a reciprocal and competitive interaction between multimodal and unimodal processing underlies stream/bounce perception with sound^12^. Similarly, a transcranial magnetic stimulation (TMS) study claims that the effect of sound on stream/bounce perception stems from audiovisual integration, not from a lack of attention^13^. Taken together, past studies appear to illustrate that audiovisual integration in higher level processing can bias stream/bounce perception toward bouncing.

Besides auditory cues, visual and tactile cues have been shown to affect stream/bounce perception. Both transient flash and tactile stimulations increase bounce perception^14,15^, suggesting that it is the result of attentional capture by the visual or tactile cue. More specifically, the lack of attentional resources for moving objects causes observers to have difficulty in tracking such objects. Furthermore, observers’ voluntary actions and their hand position appear to modulate stream/bounce perception^16,17^. An investigation^16^ into whether mouse movement during observation could influence stream/bounce perception, and found that mouse movement increased stream perception when the moving direction was congruent with the direction of one of the moving objects. A hand posture with the palms together biased individuals’ perception toward bouncing^17^. These studies indicate that factors related to physical collision serve as cues for stream/bounce perception.

Given this background, we wondered whether factors associated with psychological conflict might also affect stream/bounce perception. Physical collisions are often used as a figurative expression for psychological conflict. For example, when someone says, ‘I collided with my boss yesterday’, we tend to interpret this as engaging in psychological conflict, rather than physical collision. Moreover, psychological collisions are often accompanied by negative emotions such as anger. For instance, when individuals experience conflict with their friends, they tend to experience a negative mood, or at the very least do not feel happy. Thus, we suspected that the cues associated with emotional states might alter stream/bounce perception.

Relatedly, mental imagery has been shown to alter stream/bounce perception. In Berger and Ehrsson^18^, observers were asked to imagine a transient sound before, during, or after the coincidence of the objects. Their results showed that the proportion of trials in which participants perceived bouncing increased only when the observers imagined a transient tone at the point of coincidence. Moreover, the effect of imagery varied according to sound characteristics^19^. These studies clearly indicate that imagined sounds associated with physical collisions are sufficient to serve as cues for bouncing. In other words, it appears that a physical sound related to a collision is not necessary to influence stream/bounce perception. Considering this, we hypothesized that an emotion-related additional cue might also alter stream/bounce perception.

In this study, we investigated the effect of an emotional cue on stream/bounce perception, and used emoticons as the emotional cues. Emoticons are pictorial representations of facial expressions using punctuation marks, numbers, and letters (e.g. ‘(°∀°)’ or ‘;-(’). They are widely used in text-based communication, and can help individuals understand the magnitude and valence of emotion^20,21^. Moreover, an event-related potential (ERP) study revealed that preattentive behavioural and neural responses to emoticons were similar to those of real faces^22^, indicating that the emotional information of emoticons is processed in the manner of real faces.

There are two reasons for our use of emoticons as cues. First, emoticons can be presented near moving objects in the stream/bounce display. If we use standardized pictures or real faces as emotional cues^23,24^, these might be too large to be presented near the moving objects, and part of them would be much removed from the moving objects. Thus, observers might not see their full appearance well while they observe the moving objects. In contrast, emoticons are horizontally long, meaning that they take up little space when placed near objects moving horizontally; note that the horizontal motion was used in many previous studies^5,7,8^. Therefore, emoticons were deemed more appropriate to present with the stream/bounce display than other types of visual stimuli. Second, we can control the presentation duration of an emotional cue by using a visual stimulus. Although there is a database of standardized emotional sounds^25^ the intensity peak differs according to sound, and their durations are too long to be presented simultaneously with the stream/bounce display. For these reasons, we adopted emoticons as emotional cues.

In Experiment 1, emoticons representing anger, a sober face, and a smile were presented to examine whether facial expressions represented by emoticons influence stream/bounce perception. As described above, anger is often associated with psychological conflict, so we hypothesized that the emoticon representing anger would bias the perception toward bouncing more than would the emoticons representing the sober face and smile.

## Results and Discussions

### Experiment 1: Expression

**Figure 1:**
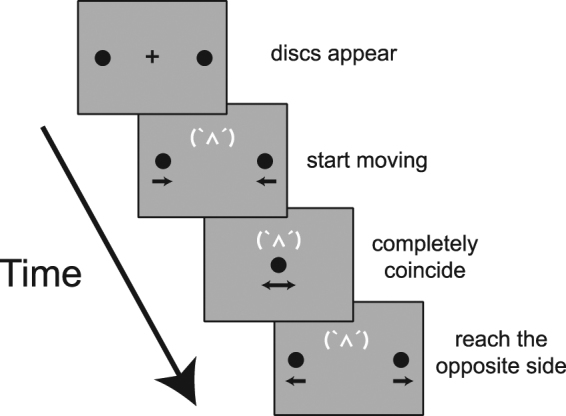
Schematic illustration of a trial in Experiment 1. Two identical black discs started moving simultaneously to the disappearance of the fixation, moved towards each other, coincided at the centre, and continued to the opposite sides of the screen, after which they vanished. The emoticon cue was presented while the objects moved. The small black arrows represent the motion directions of the black discs. Participants were asked to report their perceptual outcomes via a button-press.

**Figure 2:**
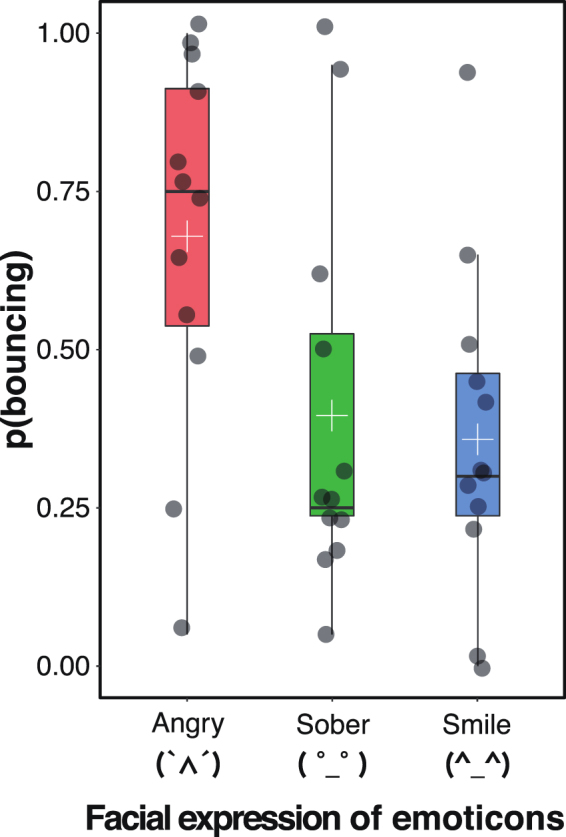
The results of Experiment 1. The dark gray circle on the boxplot represents individual data point. The white cross on the boxplot means the average response proportion of perceiving bouncing.

To investigate the effect of emoticons on stream/bounce perception, we presented the emoticons representing anger, a sober face, and a smile while the two identical discs were moving and asked observers to judge whether the discs streamed through or bounced off each other (Fig. 1). The proportion of trials in which the participants perceived ‘bouncing’ was used as the dependent variable. We conducted a one-way analysis of variance (ANOVA), which showed a significant main effect of emoticon (*F*(2, 22) = 9.01, *p* = 0.001, η_*p*_^2^ = 0.45; Fig. 2). Multiple comparisons revealed that the proportion of trials that the participants perceived ‘bouncing’ was significantly higher in the anger emoticon condition than in the sober face and smile conditions (*p*s < 0.01).

These results clearly showed that the anger emoticon biased participants’ perception toward bouncing when compared to the emoticons representing the smile and sober face. This indicates that an additional cue associated with emotional states served as a cue for bouncing during stream/bounce perception. However, emotions can be decomposed into two dimensions—valence and arousal^26^—and it remains unclear which influences stream/bounce perception. In the supplementary study (see Supplemental information), we investigated the valence and arousal of the emoticons used in Experiment 1. This showed that the emoticon representing anger (i.e. ‘(`∧´)’) had both more negative valence and higher arousal than did the emoticons representing the smile and sober face. Thus, we cannot determine whether the results of Experiment 1 stemmed from the differences in arousal, valence, or other characteristics (e.g. facial expression) of the emoticons. Therefore, in Experiments 2 and 3, we examined the effect of the valence and arousal of the emoticons on stream/bounce perception.

### Experiment 2: Arousal

**Figure 3:**
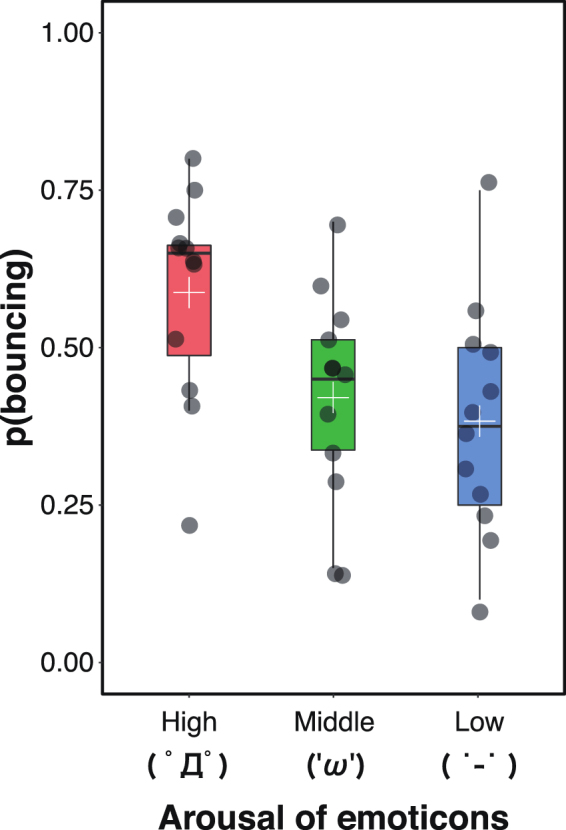
The results of Experiment 2. The dark gray circle on the boxplot represents individual data point. The white cross on the boxplot means the average response proportion of perceiving bouncing.

In this experiment, we used ‘(°Д°)’, ‘('ω')’ and ‘(˙-˙)’ as emoticons, terming them as the high-, middle-, and low-arousal emoticons, respectively. As Experiment 1, the proportion of trials in which the participants perceived ‘bouncing’ was used as the dependent variable. We conducted a one-way ANOVA, which showed a significant main effect of emoticon (*F*(2, 22) = 6.16, *p* = 0.008, η_*p*_^2^ = 0.36; Fig. 3). Multiple comparisons revealed that the proportion of trials in which ‘bouncing’ was perceived was significantly higher in the high arousal condition than in the other two conditions (*p*s < 0.05).

These results showed that stream/bounce perception is biased toward bouncing when the emoticons have higher arousal. In Experiment 3, we examined the effect of valence.

### Experiment 3: Valence

**Figure 4:**
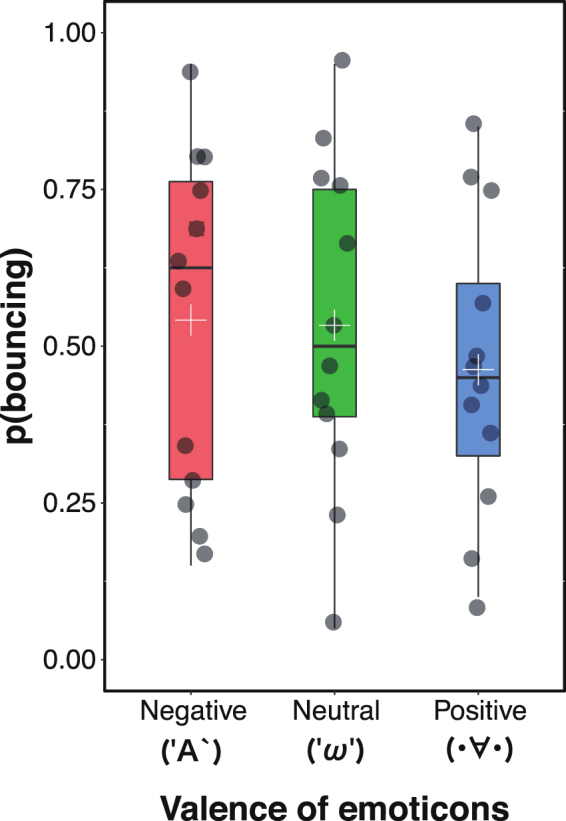
The results of Experiment 3. The dark gray circle on the boxplot represents individual data point. The white cross on the boxplot means the average response proportion of perceiving bouncing.

In this experiment, we used ‘('A`)’, ‘('ω')’, and ‘(· ∀ ·)’ as the emoticon stimuli, which corresponded the negative, neutral, and positive conditions, respectively. As Experiments 1 and 2, the proportion of trials in which the participants perceived ‘bouncing’ was used as the dependent variable. We conducted a one-way ANOVA, which showed no significant main effect of emoticon (*F*(2, 22) = 0.70, *p* = 0.51, η_*p*_^2^ = 0.06; Fig. 4).

These results revealed that stream/bounce perception is not biased by the valence of the emoticons. In other words, based on the results from Experiments 1 to 3, we might conclude that the arousal of the emoticons plays an important role in biasing stream/bounce perception.

So why does an emoticon’s arousal level bias perception toward bouncing? One explanation is that the emoticons with high arousal evoke mental imagery associated with a thumping heart in the observer, and this image of a psychological ‘bouncing’ (as with a heart) is then used as a cue for bouncing. However, there are also two alternative possibilities to explain these results. First, emoticons with high arousal might capture observers’ attention. It has been argued that the lack of attentional resources for moving objects increases the proportion of trials in which bouncing is perceived^14,15^. Moreover, visual stimuli with high arousal levels attract greater attention^27,28^. Considering these previous findings, the difference in the degree of captured attention might underlie the effect of emoticons on stream/bounce perception. Second, the results might be due to response bias: Observers might respond with ‘bouncing’ merely when a particular emoticon is presented, irrespective of their actual perception of the moving objects.

In Experiments 4 and 5, we examined these alternative possibilities. In Experiment 4, emoticon cues appeared only before the objects started moving (i.e. priming). If attentional capture by emoticons with high arousal was the cause for the biased bouncing perception, there would be no difference in the proportion of trials in which bouncing was perceived between emoticons. In Experiment 5, emoticon cues were presented 200 ms after the coincidence of the moving objects (i.e. out of the range of the temporal window^6^), although they still vanished with the moving objects. If response bias was responsible for the higher proportion of bouncing in the high-arousal condition, this same effect would be observed even when they are presented after the coincidence.

### Experiment 4: Priming

In this experiment, one of the three emoticons used in Experiment 2 was presented simultaneously with the onset of the discs, and disappeared when the discs started moving. As in other experiments, the proportion of trials in which the participants perceived ‘bouncing’ was used as the dependent variable. We conducted a one-way ANOVA, which showed a significant main effect of emoticon (*F*(2, 22) = 6.30, *p* = 0.007, η_*p*_^2^ = 0.36). Multiple comparisons revealed that the proportion of trials in which ‘bouncing’ was perceived was significantly higher in the high-arousal condition than in the other conditions (*p*s < 0.05). These results replicated those of Experiment 2, indicating that the effect of the emoticon with higher arousal does not stem from attentional capture.

### Experiment 5: Presentation after coincidence

In this experiment, we used the same three emoticons as in Experiment 2. Unlike in Experiment 2, here the emoticons were presented 200 ms after the coincidence of the discs, and disappeared along with the discs. As other experiments, the proportion of trials in which the participants perceived ‘bouncing’ was used as the dependent variable. We conducted a one-way ANOVA, which showed a significant main effect of emoticon (*F*(2, 22) = 1.60, *p* = 0.23, η_*p*_^2^ = 0.13). These results indicated that the effect of the high-arousal emoticon is not due to the response bias.

## General Discussion

We investigated the effect of emoticons on stream/bounce perception in five experiments. In Experiment 1, we manipulated the facial expressions of emoticons and found that the emoticon representing anger biased the perception toward bouncing when compared to the sober face and smile, thus supporting our hypothesis. In Experiments 2 and 3, we investigated whether arousal or valence, respectively, was the origin of the effect. These two experiments revealed that the proportion of trials in which bouncing was perceived increased with the arousal of the emoticon, whereas the valence of the emoticon had no effect on stream/bounce perception. In Experiment 4, we examined whether the results in Experiment 2 were based on attentional capture by the high-arousal emoticon, and the results discounted this possibility. In Experiment 5, we investigated the possibility of response bias for the effect of emoticons, and this possibility was also discounted. Taken together, the results of these experiments clearly suggest that emoticons can influence stream/bounce perception, and that the arousal of the emoticons plays an important role in this. Surprisingly, this is the first study to have examined not only the effect of emoticons but also the effect of emotion on stream/bounce perception.

How does the high-arousal emoticon bias perception toward bouncing? One explanation is that imagery evoked by high-arousal emoticons is used as a cue for bouncing. As noted before, previous studies have suggested that the mental imagery of sound at the coincidence of moving objects is enough to alter stream/bounce perception^18,19^. In a similar manner, emoticons with high arousal evoke an image of a thumping heart in the observer, and this image of a psychological ‘bouncing’ (as with a heart) might elicit the perception of the physical bounce of a moving object. In other words, the mental imagery associated with physical bouncing might indirectly bias the perception of bouncing.

Another explanation is that emoticons endow moving objects with emotional properties, which increase bounce perception. When an object is attributed some property, observers’ perception of the object is affected. For example, a sense of ownership makes observers hold their attention on an object^29^; they more easily remember it^30^, and perceive the object as more favorable^31^. Furthermore, when moving objects are given particular contextual information, the spatial localization of the objects is modulated^32,33^. In a similar vein, when moving objects are given emotional properties by emoticons, observers might interpret their motion as bouncing. Further studies will be needed to investigate which explanation is more appropriate.

In Experiment 4, the high-arousal emoticon affected stream/bounce perception even before the objects started moving: in other words, a priming effect of the emoticons occurred. This result goes against the finding of a previous study that the effect of sound on stream/bounce perception lies with a window ±100 ms from the complete coincidence^6^. So why does the effect of the emoticon persist even when it appeared significantly prior to the collision? We speculate that this is because emoticons represent an emotional ‘state’, not some specific ‘moment’. Emotional states are persistent to some extent, and thus the effect might be robust even for some time after presentation. On the other hand, the auditory stimuli used in previous studies clearly represented the moment of physical collision (e.g. a collision of billiard balls)^5–8^, and thus their effect might be eliminated when presented more than 100 ms before the collision. Therefore, we postulate that the temporal window in which an additional cue affects stream/bounce perception varies with what the cue represents.

Which process is affected by the emoticons: the sensory process or the decision process? Previous studies using SDT have suggested that a transient sound influences the higher-level decisional process^9,10^ (but see^11^). Furthermore, previous studies in neuroscience have reported that the origin of this effect of a transient sound is audio-visual binding^12,13^. If unimodal binding between emoticons and moving objects occurred in the same manner as for these past findings, then we might predict that emoticons would bias stream/bounce perception in the decisional process. In further studies, we need to investigate this issue closely by using neuroscientific methods (e.g. TMS).

There are some points requiring further investigation in future research. First, we must clarify whether the effect of an emotional cue on stream/bounce perception is limited to visual stimuli such as emoticons. Emotional stimuli can be presented not only visually but also auditorily. If mental imagery evoked by an emotional cue directly influences stream/bounce perception, the effect of the emotion on stream/bounce perception would occur regardless of the emotional cue’s modality. On the other hand, if the emotional property of the moving objects assigned by an emotional cue plays an important role, then the degree to which the cue biases stream/bounce perception might depend on the modality. Second, when both a physical bounce-related cue and an emotional cue are presented, would the biased perception toward bouncing still occur? Studies investigating how stream/bounce perception was affected when both visual and auditory cues were presented manipulated the luminance of moving objects at the coincidence, duration of pause at the coincidence, intensity of the auditory cue at the coincidence, or the auditory timing (or some combination of these)^34^. The results showed that the bistable perception was well predicted by the weighted sum of the cues, with the visual cues being dominant. However, it remains unknown whether this predominance of visual cues would occur when either or both cues emotionally induce bouncing (i.e. had a high arousal level). Finally, we need to know the generalizability of the effect of emoticons. Previous neurophysiological studies have found that emoticons are automatically^35,36^ and characteristically^22^ processed similar to real faces, suggesting that the processing of emoticons is general. On the other hand, it has also been shown that the recognition of emoticons is culturally dependent^37,38^. Thus, it is important to confirm the findings of the present study through cross-cultural replication studies. Investigating these issues will deepen our understanding of the link between emotional processing and bistable motion processing.

## General Methods

### Ethics statement

All the experiments in this paper were approved by the Ethical Committee of Kyushu University (approval number: 2016–001) and conducted according to the principles of the Declaration of Helsinki. Written informed consent was obtained from all participants.

### Apparatus

All the experiments in this paper were implemented with Matlab (Mathworks: Natick, MA) using the Psychophysics Toolbox extension^39,40^. The software was run on a Mac Pro computer connected to a 22′′ CRT monitor (Mitsubishi, RDF221S) with a resolution of 1024 × 768 pixels and a refresh rate was 100 Hz.

### Moving stimuli

Moving stimuli were two identical black discs (1.00° in diameter) that were presented on a grey square (14.20° × 14.20°, 49.67 cd/m^2^) whose centre was identical to the centre of the screen. The initial positions of the discs were to the left and right, respectively, to the virtual horizontal line of the centre, and the initial distance between the discs was 12.20°. They horizontally moved towards opposite sides of the screen via uniform rectilinear motion (6.21 deg/s), coinciding completely at the centre of the screen. Each disc stopped moving and then disappeared at the other disc’s starting point. The duration of their motion was 1989 ms.

### Experiment 1

#### Participants

Twelve Japanese adults (4 males, 8 females; mean age = 25.25) participated in this experiment. All the participants were naïve to the purpose of the experiment, and had either normal or corrected-to-normal vision.

#### Emoticon stimuli

We used ‘(`∧´)’, ‘(°_°)’ and ‘(^_^)’ as emoticon cues, representing anger, sober face (as a control condition), and a smile, respectively. These emoticons are primarily used in Japan. The font was Hiragino Kaku Gothic Pro, and the font size was 25 pt. The color was white. The emoticons were displayed in white and presented 2.38° above the centre of the square. They appeared and disappeared along with the moving objects (thus, their duration was also 1989 ms).

#### Procedure

Participants viewed the display binocularly from a distance of 60 cm. Before starting the first test trial, participants received instructions on completing the experiment and performed five practice trials. Each trial began when the participant pressed the space bar. First, a fixation point was presented for 1000 ms at the centre of the screen; the two black discs appeared 500 ms after the onset of the fixation. A further 500 ms after the discs appeared, the fixation point disappeared and the emoticon cue appeared when the discs began moving towards each other (the same procedure for the disappearance of the fixation was used by Zhou *et al*.^34^). The discs eventually overlapped at the centre and then continued to the opposite sides of the screen, after which they vanished along with the emoticon cue. Figure 1 shows the full series of the experimental display. The participants were then asked to judge whether the discs streamed through or bounced off each other. Participants were presented with 20 repetitions for each of the emoticons, and the order of the trials was randomized. Therefore, each participant completed 60 experimental trials.

### Experiment 2

#### Participants

Twelve Japanese adults (5 males, 7 females; mean age = 24.42) participated in this experiment. All participants were naïve to the purpose of the experiment, and had either normal or corrected-to-normal vision.

#### Emoticon stimuli

In this experiment, we used ‘(°Д°)’, ‘('ω')’ and ‘(˙-˙)’ as emoticon cues, terming them as the high-, middle-, and low-arousal emoticons, respectively. These emoticons are also widely used in Japan. Their valence and arousal were preliminarily investigated in the supplementary study introduced in the discussion of Experiment 1. This study confirmed that arousal significantly differed between these three conditions (being highest in the high condition, followed in order by the middle and low conditions), whereas the conditions did not significantly differ in valence. The other conditions were identical to those of Experiment 1.

#### Procedure

Except for the emoticons, the procedure was identical to that of Experiment 1.

### Experiment 3

#### Participants

Twelve Japanese adults (1 male, 11 females; mean age = 22.42) participated in this experiment. All participants were naïve to the purpose of this experiment, and had either normal or corrected-to-normal vision.

#### Emoticon stimuli

We used ‘('A`)’, ‘('ω')’, and ‘(· ∀ ·)’ as emoticon cues, which corresponded to the negative, neutral, and positive conditions, respectively. As with the emoticons used in Experiments 1 and 2, these are mainly used in Japan. Their valence and arousal were preliminarily investigated in the supplementary study. This study confirmed that the valence of these conditions significantly differed (being most negative in the negative condition, followed in order by the neutral and positive conditions), while there were no significant differences in arousal. The other conditions were identical to those of Experiment 1.

#### Procedure

Except for the emoticons, the procedure was identical to that of Experiment 1.

### Experiment 4

#### Participants

Twelve Japanese adults (5 males, 7 females; mean age = 24.42) participated in this experiment. They were the same participants as in Experiment 2, and all had normal or corrected-to-normal vision.

#### Emoticon stimuli

We used the three emoticons used in Experiment 2. However, unlike in Experiment 2, they were presented simultaneously with the onset of the discs, and disappeared when the discs started moving. Hence, the duration of the emoticons was only 500 ms.

#### Procedure

Except for the onset timing and duration of emoticons, the procedure was identical to that of Experiment 2.

### Experiment 5

#### Participants

Twelve Japanese adults (4 males, 8 females; mean age = 23.25) participated in this experiment. All participants were naïve to the purpose of the experiment, and had either normal or corrected-to-normal vision.

#### Emoticon stimuli

We again used the same three emoticons as in Experiment 2. Unlike in Experiment 2, the emoticons were presented 200 ms after the coincidence of the discs, and disappeared along with the discs. Hence, the duration of the emoticons was 794.50 ms.

#### Procedure

Except for the onset timing and duration of the emoticons, the procedure was identical to that of Experiment 2.

## Electronic supplementary material

Supplemental material
