# Supplementary material for: Arousing emoticons edit stream/bounce perception of objects moving past each other
Source: Sci Rep. 2018 Apr 10;8:5752. doi: 10.1038/s41598-018-23973-4 (PMC5893621; doi:10.1038/s41598-018-23973-4)
Supplement: Supplementary file 1 — Supplemental material [file 41598_2018_23973_MOESM1_ESM.docx]

**Supplemental material for**

**‘Arousing emoticons edit stream/bounce perception of objects moving past each other’**

Akihiko Gobara, Naoto Yoshimura, and Yuki Yamada

**Introduction**

In this document, we describe the pilot study in which we investigated the valence and arousal of the emoticons used in the experiments of our main study.

**Methods**

Participants.

Fifty-four Japanese adults (35 males and 19 females, mean age = 39.96) participated in this pilot study. All participants were naïve to the purpose of the research. They were recruited on Yahoo! Crowdsourcing.

Stimuli.

The emoticon stimuli used were as follows: in Experiment 1, we used ‘(`∧´)’, ‘(°_°)’, and ‘(^_^)’. For Experiments 2 to 5, we selected from among the following by the rating scores of their valence and arousal: ‘( ﾟДﾟ)’, ‘( ˙-˙ )’, ‘('A`)’, ‘(･∀･)’, ‘('ω')’, ‘( ´∀｀)’, ‘(-＿-)’ and ‘(；Д；)’. The emoticons of ‘( ´∀｀)’, ‘(-＿-)’ and ‘(；Д；)’ were not used because they did not have neither middle arousal nor neutral valence and were thus inappropriate for those experiments. All these emoticons are mainly used in Japan.

Procedure.

The pilot study was conducted on the Web using Google Forms. All questionnaire contents were written in Japanese. In the first section, participants were asked to indicate their sex and age. In the second section, they rated the valence and arousal of each emoticon using a nine-point Likert scale (valence: 1 = extremely positive, 9 = extremely negative; arousal: 1 = feel extremely excited, 9 = do not feel excited at all).

**Results and discussion**

In the analysis, we inverted the rating scores for arousal (such that 1 = do not feel excited at all, 9 = feel extremely excited), and then used valence and arousal ratings as dependent variables. The ratings of valence and arousal for each emoticon are shown in Table S1. First, we conducted a one-way ANOVA on the emoticons used in Experiment 1. For valence, we observed a significant main effect (*F*(2, 104) = 135.70, *p* < .001, η*_p_*^2^ = .72). Multiple comparisons revealed that ‘(`∧´)’ was rated as significantly more negative than ‘(°_°)’ and ‘(^_^)’, while ‘(°_°)’ was more negative than ‘(^_^)’ (*p*s < .001). For arousal, there was also a significant main effect (*F*(2, 104) = 37.15, *p* < .001, η*_p_*^2^ = .42), with ‘(`∧´)’ being rated as having significantly higher arousal than ‘(°_°)’ or ‘(^_^)’ (*p*s < .001). These results suggest that the emoticon representing anger was both more negative and had higher arousal compared to the emoticons representing the sober face and smile.

Next, we selected ‘( ﾟДﾟ)’, ‘( ˙-˙ )’ and ‘('ω')’ as the emoticon stimuli for Experiments 2, 4, and 5, and conducted a one-way ANOVA. The results showed that there was a significant main effect of emoticon for arousal (*F*(2, 104) = 46.24, *p* < .001, η*_p_*^2^ = .47) but not for valence (*F*(2, 104) = 2.14, *p* = .12, η*_p_*^2^ = .04). Multiple comparisons for arousal showed that ‘( ﾟДﾟ)’ had higher arousal than ‘( ˙-˙ )’ and ‘('ω')’, while ‘('ω')’ had higher arousal than ‘( ˙-˙ )’ (*p*s < .001). Therefore, the emoticons in Experiments 2, 4, and 5 successfully manipulated arousal while controlling valence.

Finally, we selected ‘('A`)’, ‘(･∀･)’, and ‘('ω')’ as the emoticon stimuli for Experiment 3, and conducted a one-way ANOVA. The results were the opposite of those of the previous one mentioned above: There was a significant main effect of emoticon for valence (*F*(2, 104) = 32.62, *p* < .001, η*_p_*^2^ = .39) but not for arousal (*F*(2, 104) = 1.42, *p* = .25, η*_p_*^2^ = .03). Multiple comparisons for valence showed that ‘('A`)’ was rated as more negative than ‘(･∀･)’ and ‘('ω')’, while ‘('ω')’ was more negative than ‘(･∀･)’ (*p*s < .001). These results indicate that the emoticons in Experiment 3 successfully manipulated valence while controlling for arousal.

**Table S1**. The results of the pilot study
